# Insect-Specific Flaviviruses: A Systematic Review of Their Discovery, Host Range, Mode of Transmission, Superinfection Exclusion Potential and Genomic Organization 

**DOI:** 10.3390/v7041927

**Published:** 2015-04-10

**Authors:** Bradley J. Blitvich, Andrew E. Firth

**Affiliations:** 1 Department of Veterinary Microbiology and Preventive Medicine, College of Veterinary Medicine, Iowa State University, Ames, IA 50011, USA; 2 Department of Pathology, University of Cambridge, Cambridge CB2 1QP, UK; E-Mail: aef24@cam.ac.uk

**Keywords:** flavivirus, insect-specific, host range, transmission, genomic organization

## Abstract

There has been a dramatic increase in the number of insect-specific flaviviruses (ISFs) discovered in the last decade. Historically, these viruses have generated limited interest due to their inability to infect vertebrate cells. This viewpoint has changed in recent years because some ISFs have been shown to enhance or suppress the replication of medically important flaviviruses in co-infected mosquito cells. Additionally, comparative studies between ISFs and medically important flaviviruses can provide a unique perspective as to why some flaviviruses possess the ability to infect and cause devastating disease in humans while others do not. ISFs have been isolated exclusively from mosquitoes in nature but the detection of ISF-like sequences in sandflies and chironomids indicates that they may also infect other dipterans. ISFs can be divided into two distinct phylogenetic groups. The first group currently consists of approximately 12 viruses and includes cell fusing agent virus, Kamiti River virus and Culex flavivirus. These viruses are phylogenetically distinct from all other known flaviviruses. The second group, which is apparently not monophyletic, currently consists of nine viruses and includes Chaoyang virus, Nounané virus and Lammi virus. These viruses phylogenetically affiliate with mosquito/vertebrate flaviviruses despite their apparent insect-restricted phenotype. This article provides a review of the discovery, host range, mode of transmission, superinfection exclusion ability and genomic organization of ISFs. This article also attempts to clarify the ISF nomenclature because some of these viruses have been assigned more than one name due to their simultaneous discoveries by independent research groups.

## 1. Introduction

All viruses in the genus *Flavivirus* (family *Flaviviridae*) possess a single-stranded, positive-sense RNA genome of approximately 11 kb [1]. The genome usually encodes a single open reading frame (ORF) that is flanked by 5' and 3' untranslated regions (UTRs) of ~100 and ~400–700 nt, respectively [2]. The ORF encodes a large polyprotein that is co- and post-translationally cleaved to generate three structural proteins, designated the capsid (C), premembrane/membrane (prM/M) and envelope (E) proteins, and seven nonstructural (NS) proteins in the gene order: 5'–C–prM(M)–E–NS1–NS2A–NS2B–NS3–NS4A–2K–NS4B–NS5-3' [1,3]. The genomes of some flaviviruses appear to encode an additional protein as a consequence of ribosomal frameshifting as discussed later in this review. 

**Figure 1:**
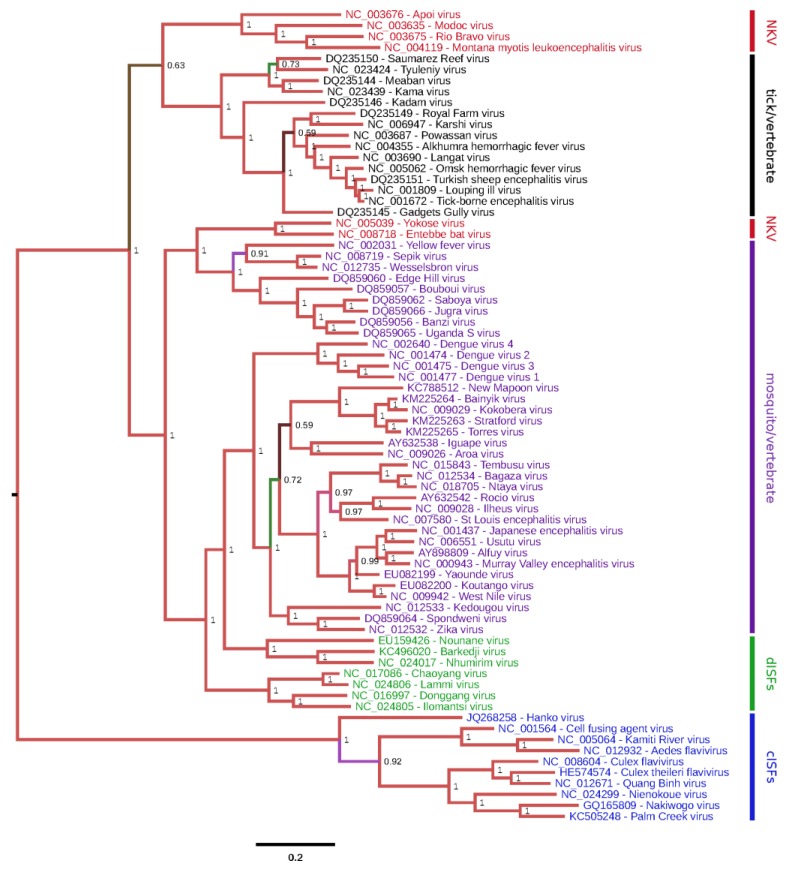
Phylogenetic tree for genus *Flavivirus*. Complete polyprotein amino acid sequences were aligned using MUSCLE [14]. Regions of ambiguous alignment were excised using Gblocks [15] with default parameters, after which 1774 amino acid positions were retained. A maximum likelihood phylogenetic tree was estimated using the Bayesian Markov chain Monte Carlo method implemented in MrBayes version 3.2.3 [16] sampling across the default set of fixed amino acid rate matrices, with 10 million generations, discarding the first 25% as burn-in. The figure was produced using FigTree (http://tree.bio.ed.ac.uk/software/figtree/). The tree is midpoint-rooted, nodes are labelled with posterior probability values, and branches are also highlighted with alternative colors. Species names are color-coded as follows: cISFs—blue; dISFs—green; NKV flaviviruses—red; mosquito/vertebrate flaviviruses—purple; tick/vertebrate flaviviruses—black.

Despite their similar genomic organizations, flaviviruses possess fundamental differences in their host ranges and transmissibilities. Most recognized flaviviruses are transmitted horizontally between hematophagous arthropods and vertebrate hosts and are therefore considered to be dual-host viruses. Dual-host flaviviruses can be further divided into mosquito/vertebrate and tick/vertebrate viruses. Examples of mosquito/vertebrate flaviviruses include dengue virus (DENV), yellow fever virus, Japanese encephalitis virus (JEV) and West Nile virus (WNV), all of which are human pathogens of global concern [4]. Flaviviruses of localized public health concern include St Louis encephalitis virus (SLEV) and Murray Valley encephalitis virus (MVEV). Tick/vertebrate flaviviruses associated with serious human disease include tick-borne encephalitis virus, Langat virus and Powassan virus. Not all flaviviruses cycle between arthropods and vertebrates; some have a vertebrate-specific host range while others appear to be insect-specific. Vertebrate-specific flaviviruses, also known as No Known Vector (NKV) flaviviruses, can be divided into two groups: those isolated exclusively from rodents (e.g., Modoc virus; MODV) and those isolated exclusively from bats (e.g., Rio Bravo virus) [5,6]. It has been suggested that NKV flaviviruses are maintained in nature by horizontal transmission among hosts [7,8,9,10]. Insect-specific flaviviruses (ISFs) can also be divided into two distinct groups (Figure 1). ISFs in the first group are phylogenetically distinct from all other known flaviviruses and, for the purpose of this review, will be referred to as classical ISFs (cISFs) since they were discovered first. ISFs in the second group phylogenetically affiliate with mosquito/vertebrate flaviviruses and, for the purpose of this review, will be referred to as dual-host affiliated ISFs (dISFs). This group is apparently not monophyletic. There has been a dramatic increase in the number of ISFs discovered in the last ten years and this is partly due to advances in the methods available for virus detection. 

In addition to the growing number of ISFs recently described, cISF-like sequences, designated cell silent agent (CSA), have been found integrated in the genomes of *Aedes* spp. mosquitoes [11,12]. One sequence was shown to encode a NS1-NS4A-like transcript. Partial E, NS4B and NS5-like sequences were also identified. The occasional integration of flavivirus sequences into the mosquito genome could occur due to the reverse transcriptase and integrase activities of co-infecting or endogenous retroviruses, and indeed the integration of viral sequences into the host genome has since been documented for many other RNA viruses and host species [13]. Most integrated sequences are highly fragmented or have internal stop codons but several encode intact ORFs. Importantly, some genome-integrated sequences may be transcribed and therefore, the detection of flavivirus-like RNA in an organism is not necessarily proof that the organism carries an active flavivirus infection.

## 2. Classical Insect-Specific Flaviviruses

### 2.1. Discovery, Geographic Distribution and Natural Host range

Classical ISFs have a ubiquitous geographic distribution; these viruses have been isolated from mosquitoes in every continent with the exception of Antarctica (Table 1). Additionally, cISF-like sequences have been detected in sandflies and midges in several Mediterranean countries as discussed later in this section. The first cISF to be discovered was cell fusing agent virus (CFAV) after its isolation from *Aedes aegypti* cell cultures over 40 years ago [17]. This discovery received little attention as illustrated by the fact that 17 years passed before another article on the virus was published [18]. CFAV has since been isolated from or detected in field-collected mosquitoes in Indonesia [19], Mexico [20], Puerto Rico [21] and Thailand [22,23]. CFAV has also been isolated from laboratory colonies originally established from mosquitoes collected in the United States [24]. Additionally, CFAV-like sequences have been identified in field-collected mosquitoes in Argentina (Genbank Accession No. DQ335466, DQ335467 and DQ431718) but the sequences are too short (87–110 nt) for reliable analysis.

**Table 1:** Geographic distribution and natural host range of classical insect-specific flaviviruses.

^a^ Virus	Isolate Available	Geographic Distribution	Natural Host Range	References
Aedes flavivirus (AEFV)	Yes	Japan (2003), Italy (2008), USA (2011), ^b^ Thailand (2012)	*Ae. albopictus, Ae. flavopictus, Cx. pipiens*	[12,19,24,25,26,27]
Aedes galloisi flavivirus (AGFV)	Yes	Japan (2003)	*Ae. galloisi*	[28]
Calbertado virus (CLBOV)	Yes	Canada (2003), USA (2006)	*Cx. tarsalis, Cx. pipiens*	[29,30]
Cell fusing agent virus (CFAV)	Yes	Laboratory (1975), Puerto Rico (2002), Indonesia (2004), Mexico (2007), Thailand (2008), ^b^United States (2012)	*Ae. albopictus*, *Ae. aegypti*, *Culex* spp.	[17,19,20,21,22,24]
Culex flavivirus (CxFV)	Yes	Japan (2003), Indonesia (2004), China (2006), Guatemala (2006), USA (2006), Mexico (2007), Trinidad (2008), Uganda (2008), Argentina (2009)	*Cx. interrogator, Cx. maxi, Cx. nigripalpus, Cx. pipiens, Cx. quinquefasciatus, Cx. restuans, Cx. tarsalis, Cx. tritaeniorhynchus, Cx. usquatus*	[29,31,32,33,34,35,36,37,38]
^c^ Culex theileri flavivirus (CTFV)	Yes	Spain (2006), Portugal (2009–2010), Greece (2010), Thailand (date not specified)	*Cx. fuscocephala, Cx. pipiens, Cx. theileri*	[25,39,40,41] (Genbank Accession No. AY457040)
^d^ Hanko virus (HANKV)	Yes	Finland (2005), Spain (2006), Italy (ca. 2007), Portugal (ca. 2007)	*Ae. caspius, Ae. detritus, Ae. vexans, Cx. pipiens, Cx. perexiguus, Cx. theileri*	[25,39,42,43]
Kamiti River virus (KRV)	Yes	Kenya (1999)	*Ae. macintoshi*	[44,45]
Nakiwogo virus (NAKV)	Yes	Uganda (2008)	*Mansonia africana nigerrima*	[38]
^e^ Nienokoue virus(NIEV)	^f^ Yes	Cote d’Ivoire (2004)	*Culex* spp.	(Genbank Accession No. NC_024299)
Palm Creek virus (PCV)	Yes	Australia (2010)	*Coquillettidia xanthogaster*	[46]
Quang Binh virus (QBV)	Yes	Vietnam (2002), China (2009)	*An. sinensis, Cx. tritaeniorhynchus*	[47,48,49]

^a^ This table is restricted to viruses for which more than 300 nt of sequence data are available; ^b^ AEFV and CFAV were isolated from laboratory colonies established in 2012 from mosquitoes collected in Thailand and the U.S.A, respectively; ^c^ Also known as Spanish Culex flavivirus (SCxFV) and Wang Thong virus (WTV); ^d^ Also known as Ochlerotatus flavivirus (OcFV), Spanish Ochlerotatus flavivirus (SOcFV) and Ochlerotatus caspius flavivirus from Portugal (OCFV_PT_); ^e^ An acronym has not been assigned to Nienokoue virus and therefore, for the purpose of this review, NIEV will be used; ^f^ Information provided in the Genbank database implies that an isolate is available for NIEV.

The next cISF to be discovered was Kamiti River virus (KRV) after its isolation from mosquitoes in Kenya in 1999 (Table 1) [44,45]. A KRV-like sequence has also been detected in mosquitoes in Argentina (Genbank Accession No. DQ335465) but, due to the limited amount of sequence data obtained in the study (a 124-nt region of the NS5 gene was sequenced), additional information is needed to determine whether KRV occurs in this country. The third member of the cISF group to be reported was Culex flavivirus (CxFV). The virus was first isolated from *Culex* spp. mosquitoes in Japan and Indonesia in 2003–2004 [31] and later isolated from *Culex* spp. mosquitoes in Argentina [32], Brazil [50], China [33,51], Guatemala [34], Mexico [35,36,52], Taiwan [53], Trinidad [37], Uganda [38] and the United States [29,37,54,55,56]. 

In the last ten years, several other cISFs have been described and these include: Aedes flavivirus (AEFV) in Japan [19], Italy [12,25,57], Thailand [24] and the United States [26], Aedes galloisi flavivirus (AGFV) in Japan [28], Calbertado virus (CLBOV) in Canada [30,58] and the USA [29], Nakiwogo virus (NAKV) in Uganda [38], Nienokoue virus (NIEV) in Cote d’Ivoire (Genbank Accession No. NC_024299), Palm Creek virus (PCV) in Australia [46] and Quang Binh virus (QBV) in Vietnam [47] and China [48] (Table 1). Several other novel cISFs have also been identified. However, after performing nucleotide sequence alignments, it is apparent that some of these viruses have been assigned multiple names due to their simultaneous discoveries by independent research groups as discussed below. 

Three apparently novel cISFs were reported in the literature within in space of a few months: Hanko virus (HANKV) after its isolation from *Ae. caspius* in Finland in 2005 [42], Ochlerotatus flavivirus (OcFV) after its isolation from various *Aedes* and *Culex* spp. mosquitoes in Spain, Italy and Portugal from 2007 to 2010 [25] and Spanish Ochlerotatus flavivirus (SOcFV) after its isolation from *Ae. caspius* in Spain in 2006 [39] (Table 1). Huhtamo and colleagues sequenced the entire ORF of their virus [42]; the two other research groups sequenced a 238 to 917 nt region of the NS5 gene [25,39]. Pairwise nucleotide sequence alignments of the 163-nt region shared by representative HANKV, OcFV and SOcFV sequences (Genbank Accession Nos., JQ268258, GQ476991 and JF707790 respectively) revealed that these viruses are 91% to 96% identical. It has been proposed that flaviviruses with >84% nucleotide sequence identity should be classified within the same species [59]. Although these alignments were performed using short sequences in the relatively highly conserved NS5 region, according to the above criterion, HANKV, OcFV and SOcFV are likely to be the same virus species. For the remainder of this review, the virus will be referred to as HANKV because Huhtamo and colleagues [42] performed the most comprehensive sequence analysis. The following year, an article describing an apparently novel cISF designated Ochlerotatus caspius flavivirus from Portugal (OCFV_PT_) was published [43]. The authors sequenced almost all of the OCFV_PT_ genome and reported that it has 89% nucleotide identity to the corresponding region of HANKV. In accordance to the criterion for flaviviral species demarcation [59], this virus is HANKV and not an unrecognized cISF species.

Another cISF has been assigned multiple names: Culex theileri flavivirus (CTFV or CxthFV), Spanish Culex flavivirus (SCxFV) and Wang Thong virus (WTV). Culex theileri flavivirus (CTFV) was isolated from *Cx. theileri* in Portugal in 2009–2010 [40] (Table 1). The same virus was independently discovered by another research group after its isolation from *Cx. theileri* in Portugal and Spain in 2007–2010, and coincidently given the same name but different acronym (e.g., CxthFV) [25]. SCxFV is the name assigned to several isolates obtained from *Cx. theileri* and *Cx. pipiens* in Spain in 2006 [39]. WTV was detected in *Cx. fuscocephala* in Thailand on an unspecified date (Genbank Accession No. AY457040). Parreira and colleagues sequenced almost the entire genome of their virus [40]; the other groups sequenced a 159 to 917 nt region of the NS5 gene [25,39]. Pairwise sequence alignments of the 140-nt region shared by representative CTFV, CxthFV, SCxFV and WTV sequences (Genbank Accession Nos. HE574574, EU716420, JF707811 and AY457040, respectively) revealed that these viruses are 91% to 100% identical. Therefore, according to the criterion for flaviviral species demarcation [59], CTFV, CxthFV, SCxFV and WTV are the same virus. For the remainder of this review, the virus will be referred to as Culex theileri flavivirus since this name was chosen by two of the four research groups that made the discovery [25,40]. The acronym selected by Parreira and colleagues (e.g., CTFV) will be used because these researchers performed the most comprehensive sequence analysis [40].

Quang Binh virus or a novel QBV-like virus (designated Yunnan Culex flavivirus; YNCxFV) was isolated from 10 pools of *Cx. tritaeniorhynchus* and one pool of *Anopheles sinensis* collected in the Yunnan Province of China in 2009 [48] (Table 1). The genome of one isolate was completely sequenced and the ORF was reported to have 83.0% nucleotide identity to the corresponding region of the prototypical QBV isolate. Because this figure is close to the >84% value for flavivirus species demarcation [59], the authors opted for a conservative approach and considered their isolate to be a strain of QBV [48]. The authors also pointed out that their isolate was obtained from the same mosquito spp. and geographic region as QBV (Yunnan Province borders Vietnam) and that additional testing (*i.e.*, neutralization assays) was required before the isolate could be considered the prototypical member of a novel species. For the purpose of this review, the entire genomic sequences (as opposed to the entire ORFs) of the prototypical QBV and YNCxFV isolates (Genbank Accession Nos. FJ644291 and KC464457, respectively) were aligned to shed more light on their genetic relatedness. The two sequences have 83.7% nucleotide identity which is even closer to the threshold value establish by Kuno *et al* [59]. This analysis also revealed that the two genomes are of the exact same length (10,865 nt). Thus, YNCxFV could very well be a divergent isolate of QBV rather than a novel cISF species. A comparison of the lengths of the non-coding regions revealed that the 5' UTR of YNCxFV is one nucleotide shorter than the corresponding region of QBV (111 nt *vs*. 112 nt) while its 3' UTR is one nucleotide longer (674 nt *vs*. 673 nt). Some cISFs display strain-specific differences in the lengths of their non-coding region lengths; for example, the 5' and 3' UTRs of CxFV Toyama 1431 strain (Genbank Accession No. AB701775) are both one nucleotide shorter than the corresponding regions of CxFV Tokyo strain (Genbank Accession No. AB262759). Because it is unclear whether YNCxFV is an unusual isolate of QBV or a distinct virus species, the conservative approach opted by Zuo and colleagues [48] will be used for the remainder of this review and their virus will be referred to as QBV. 

A potentially novel cISF, designated Aedes vexans flavivirus (AeveFV), was isolated from *Ae. vexans* in Italy and the Czech Republic in 2008–2009 [25]. A short (131 to 263 nt) region of the NS5 gene was sequenced and shown to have no more than 80% nucleotide identity to the corresponding region of its closest relative, consistent with the discovery of a new virus. However, the sequences are short and more comprehensive sequencing experiments are needed to determine whether AeveFV is an unrecognized virus. Likewise, a potentially novel cISF, designated Czech Aedes vexans flavivirus (Czech AeveFV), was isolated from *Ae. vexans* in the Czech Republic in 2009 [25] but the corresponding sequences are too short (209 to 217 nt) for reliable analysis. Another potentially novel cISF, designated Aedes cinereus flavivirus (AeciFV), was detected in *Ae. cinereus* in the U.K. in 2010 [25]. Comprehensive sequence alignments were not performed (the virus was compared to only five other cISFs) and the sequence has not been deposited into the Genbank database. Additional information is needed to determine whether AeciFV is a novel cISF. Although AeveFV, Czech AeveFV and AeciFV could very well represent novel cISFs, they are not listed in Table 1; the table is restricted to viruses for which more than 300 nt of sequence data are available. 

Two 917-nt sequences corresponding to a cISF designated Culex pipiens flavivirus were detected in mosquitoes in Portugal in 2009–2010 (Genbank Accession No. HE997068-HE997069). The sequences are 97% identical to the corresponding region of CTFV and therefore, the species name of Culex pipiens flavivirus should be discontinued. Multiple 165-nt sequences corresponding to a virus denoted as Culicinae flavivirus were also identified in mosquitoes in Portugal (Genbank Accession No. EU716415–EU716419 and EU716421–EU716424). This species name should also be discontinued because these sequences are 90%–99% identical to the corresponding region of HANKV. Other species names that appear in the NCBI taxonomy and Genbank databases that should be discontinued for similar reasons are Mediterranean Culex flavivirus, Mediterranean Ochlerotatus flavivirus and mosquito flavivirus (for example, Genbank Accession No. JF707854, JF707806 and KF882513). 

Although cISFs have been isolated exclusively from mosquitoes, cISF-like sequences have been detected by molecular methods in other dipterans indicating that cISFs may not have a mosquito-restricted host range. Novel cISF-like sequences of 157 nt were detected in male *Phlebotomus perniciosus* in Algeria in 2006–2007 [60]. PCR products were not detected when the reverse-transcription step was excluded suggesting that a novel virus, rather than CSA, was identified. Virus isolation experiments were not performed because the sandflies had been preserved in guanidinium thiocyanate. Classical ISF-like sequences were also detected in sandflies in Spain but once again virus isolation experiments were not attempted [61]. In another study, a 6567-nt cISF-like sequence was identified by RNA deep sequencing in chironomids (non-biting midges) in France, although again isolation of virus particles was not attempted [62]. 

### 2.2. In Vitro and in Vivo Replication Potential in Vertebrates and Arthropod Cells

Classical ISFs have not been isolated from any vertebrates in nature and cannot replicate in any vertebrate cell lines that have been tested; thus, these viruses are assumed to possess a vertebrate-incompetent replication phenotype. The most comprehensive *in vitro* host range studies were performed with PCV which was shown to lack the ability to replicate in hamster (BHK-21), human (SW-13), monkey (Vero) and porcine (PS-EK) cells [46], and CxFV which cannot replicate in avian (DF-1), hamster (BHK-21) or monkey (Vero) cells [29,31]. Most other cISFs have been demonstrated to lack the ability to infect hamster (BHK-21) and/or monkey (Vero) cells [17,19,28,29,39,44,47]. Attempts to infect suckling mice with AeFV and CxFV by intracerebral inoculation were unsuccessful [26,37]. 

Every described cISF possesses the ability to replicate in *Ae. albopictus* (C6/36) cells [17,19,28,30,31,38,40,42,45,46,47]. Some cISFs induce cytopathic effect (CPE) and form plaques in C6/36 cells whereas others do not. Another determinant of whether CPE occurs is the passage history of the virus. PCV does not induce CPE in C6/36 cells after the first or second passage but often morphological changes (*i.e.*, syncytia and vacuolation in cells) are observed by the fourth passage [46]. Moderate CPE was periodically observed in C6/36 cells inoculated with CxFV that had been passed at least twice whereas CPE was usually absent in cells infected with the original inoculum or virus passed once [31]. CxFV isolates from Japan do not plaque in C6/36 cells [31] unlike isolates from Guatemala [63]. CxFV and CFAV both reach maximum titers of approximately 10^7^ plaque forming units (pfu)/mL in C6/36 cells while KRV produces a maximum titer of 10^8^ pfu/mL in this cell line [44,63]. The replicative potentials of select cISFs have also been assessed in other mosquito cell lines including CFAV which replicates in *Ae. albopictus* (AA23) and *Ae. aegypti* (A20) cells [21] and KRV which replicates in *Ae. pseudoscutellaris* (AP-61) and *Ae. aegypti* cells [44].

*In vivo* experiments have been performed to characterize the replicative potential and tissue tropisms of CxFV in *Culex* spp. mosquitoes [63,64]. CxFV establishes a systemic infection in *Cx. pipiens*, as indicated by the detection of viral RNA in all tissues examined (salivary glands, ovaries, testes, head, fat bodies and midguts) [64]. The presence of CxFV RNA in the salivary glands is interesting because, due to the vertebrate-incompetent replication phenotype of this virus, establishment of a salivary gland infection does not appear necessary for its persistence in nature. CxFV was not detected in the saliva of *Cx. quinquefasciatus* infected with CxFV alone but was detected in the saliva of mosquitoes co-infected with CxFV and WNV [63]. 

### 2.3. Transmission 

Vertical transmission is defined as the process by which an infected female directly transmits a pathogen to her progeny. The detection of cISFs in mosquitoes of all life stages, including adults of both sexes, indicates that vertical transmission is a major mechanism by which these viruses persist in mosquitoes in nature [21,26,29,45,52,65]. The initial isolations of KRV were made from *Ae. mcintoshi* larvae and pupae [45], CxFV RNA has been detected in *Cx. pipiens* egg rafts, larvae, adult males and adult females [29,65] and AeFV was isolated from a pool of male *Ae. albopictus* reared to adults from field-collected larvae [26]. Additionally, the first isolate of CFAV was obtained from the C6/36 cell line which was derived from *Ae. albopictus* larvae [17,66].

One mechanism of vertical transmission is transovarial transmission (TOT), defined as the process by which progeny of infected females are directly infected in the egg stage within the ovary before release and subsequent insemination. Experiments performed with field-infected *Cx. pipiens* revealed that TOT is an efficient mechanism for CxFV persistence [64]. Filial infection (FI) and TOT rates of 97.4% and 100%, respectively were reported. These values are considerably greater than the <1% FI and vertical infection rates typically reported in mosquitoes infected with dual-host flaviviruses [67,68,69]. Viral dissemination to the ovaries is necessary for TOT to occur. Accordingly, CxFV RNA was detected in the ovaries of F_1_ produced from field-infected *Cx. pipiens* [64]. Interestingly, TOT did not occur when uninfected laboratory-colonized *Cx. pipiens* were infected with CxFV by needle inoculation [64]. One explanation for the different TOT rates between the experimentally and naturally infected *Cx. pipiens* could be that mosquitoes with lifelong infections (*i.e.*, vertically infected mosquitoes) are more susceptible to TOT than mosquitoes infected as adults. The mosquitoes did not possess an ovarian infection barrier because CxFV RNA was detected in their ovaries.

Vertical transmission of KRV has been demonstrated in laboratory-colonized *Ae. aegypti* [70]. The FI rate in the F_1_ produced by the infected mosquitoes was 3.9% while the TOT rate was not reported. One likely explanation for the dramatically lower FI rate in this study as compared to the FI rate of 97.4% reported for CxFV is that there is no direct evidence to indicate the *Ae. aegypti* is a natural host of KRV. The virus has only been isolated from *Ae. macintoshi* in the field [45] and vertical transmission is presumably more efficient in the natural mosquito host. 

The contribution of venereal transmission in cISF persistence was investigated by allowing CxFV-infected male *Cx. pipiens* to mate with uninfected females [65]. Reciprocal mating experiments were also performed. Virus was transmitted to 2.4%–5.3% of the mosquitoes indicating that venereal transmission has a minor role in CxFV persistence. Horizontal transmission among larvae and non-sexual contact transmission among adults were considered unlikely modes of CxFV maintenance [65]. Efficient *per os* transmission of KRV has been reported for *Ae. aegypti* [70]. In these studies, 62.4% of mosquitoes that fed on infectious blood were positive for KRV by virus isolation in cell culture. Virus was also isolated from 90.2% of second instar larvae exposed to KRV-infected C6/36 cells. Efficient *per os* infection has also been reported for *Ae. aegypti* exposed to Eilat virus (EILV), an insect-specific alphavirus, via infectious blood meal [71]. Infection and dissemination rates of 63%–78% and 8%–26% were observed. *Ae. albopictus*, *An. gambiae* and *Cx. quinquefasciatus* were also susceptible to EILV infection, albeit at a lower rate. Studies need to be performed to assess whether efficient *per os* infection occurs in mosquitoes exposed to cISFs via natural food sources (*i.e.*, nectar). 

Some cISFs exhibit seasonal activity [29,37,52,54]. These findings could be considered unexpected if vertical transmission was the sole mechanism for their persistence in nature. CxFV was detected in mosquitoes in Texas, U.S.A from November to March but not April to August, even though mosquitoes were abundant at these times [37]. CLBOV was not detected year-round in *Cx. pipiens* and *Cx. tarsalis* in Colorado, U.S.A [29]. These findings could indicate that another mode of transmission has a major role in cISF persistence. Alternatively, these findings could be a consequence of sampling biases, small sample sizes or limitations in viral detection methods. 

### 2.4. Competitive Interaction between cISFs and Dual-Host Flaviviruses

Superinfection exclusion (or homologous interference) is the process by which host cells infected with one virus do not support productive replication of the same or similar virus [72]. This phenomenon has been observed during infections by a broad range of viruses and can occur in both vertebrate and invertebrate hosts [73,74,75,76]. Data regarding the abilities of cISFs to induce superinfection exclusion of dual-host flaviviruses in mosquito cells has been variable. Prior infection with PCV significantly reduced WNV and MVEV replication in C6/36 cells [46]. In contrast, prior exposure to CxFV had no effect on WNV replication in C6/36 cells [63]. In another study, WNV titers were significantly lower in CxFV-infected C6/36 cells compared to uninfected C6/36 cells at earlier, but not later, time points [65]. The *in vitro* growth kinetics and yields of JEV and DENV did not differ significantly in *Cx. tritaeniorhynchus* cells persistently infected with CxFV when compared to cells without pre-existing CxFV infections although JEV superinfection induced severe CPE [77]. Taken together, the above data indicate that cISFs can suppress the *in vitro* replication of dual-host flaviviruses in mosquito cells under some circumstances. 

Vector competence experiments were performed with two colonies of *Cx. pipiens*, one persistently infected with CxFV and the other not, in order to evaluate the effect of CxFV on WNV transmission in this mosquito spp. [65]. At 7 days p.i., a significantly lower percentage of CxFV-infected mosquitoes (72%) had disseminated WNV infections compared to single-virus infected mosquitoes (94%). Infection and transmission rates did not differ significantly. At 14 days p.i., WNV infection, dissemination and transmission rates did not differ significantly between the two groups. These data indicate that CxFV can suppress the *in vivo* replication of WNV early during infection. However, it should be noted that the mosquito colonies used for these experiments are from different geographic locations (Colorado and Iowa) and therefore, their differential susceptibilities to WNV infection could to due to factors other than co-infection with CxFV. In another study, sequential infection experiments demonstrated that prior infection with CxFV had no significant effect on WNV infection, dissemination or transmission in *Cx. quinquefasciatus* [63]. Coinfection experiments demonstrated that the ability of WNV to be transmitted by *Cx. quinquefasciatus* after simultaneous inoculation with WNV and CxFV was strain-specific. A significantly higher percentage of co-inoculated Honduras *Cx. quinquefasciatus* transmitted WNV compared to mosquitoes inoculated with WNV alone. In contrast, the percentage of co-inoculated Sebring *Cx. quinquefasciatus* that transmitted WNV did not differ significantly from the single-virus infected control group. These experiments indicate that cISFs can enhance the transmissibility of dual-host flaviviruses under some circumstances. In this regard, a positive ecological association between CxFV and WNV was reported in field-collected *Culex* spp. mosquitoes in Chicago, U.S.A. in 2006 [56]. WNV-positive mosquito pools were four times more likely to be positive for CxFV compared to spatiotemporally matched WNV-negative pools.

### 2.5. Genome Sequencing and Phylogeny

Complete genome sequences are available for five cISFs: AeFV, CFAV, CxFV, KRV and QBV (Table 2). The prototypical isolates of these viruses possess 5’ UTRs of 91 to 113 nt, consistent with the lengths of the 5’ UTRs of most other flaviviruses [2]. The 3’ UTRs of CFAV, CxFV and QBV are also of the expected size. However, the 3' UTRs of AeFV and KRV are unusually long. The 3' UTR of AeFV consists of 945 nt while the 3' UTR of KRV consists of 1205 nt which is approximately twice the length of a typical flavivirus 3'UTR [2]. It has been proposed that the unusually long KRV 3'UTR resulted from an almost complete duplication of a precursor sequence [78]. According to the Genbank database, the complete genome of NIEV has also been sequenced (Genbank Accession No. NC_024299). However, the 3' UTR of this virus is remarkably short (167 nt) and therefore, we consider it likely that the sequence is truncated at the 3' end. Of the remaining cISFs, complete polyprotein ORF sequences are available for CTFV, HANKV, NAKV and PCV. Limited sequence data are available for AGFV and CLBOV; 556 and 946 nt of their NS5 genes have been sequenced, respectively. 

**Table 2:** Summary of sequence data available for classical insect-specific flaviviruses.

Virus	Sequence Data Available	Length of Genome (nt)	Length of 5’ UTR (nt)	Length of 3’ UTR (nt)	^a^ Genbank Accession No.
Aedes flavivirus	Genome	11,064	96	945	NC_012932
Aedes galloisi flavivirus	Partial NS5	^b^ -	-	-	AB639347
Calbertado virus	Partial NS5	-	-	-	EU569288
Cell fusing agent virus	Genome	10,695	113	556	NC_001564
Culex flavivirus	Genome	10,834	91	657	NC_008604
Culex theileri flavivirus	ORF	-	-	-	HE574574
Hanko virus	ORF	-	-	-	JQ268258
Kamiti River virus	Genome	11,375	96	1205	NC_005064
Nakiwogo virus	ORF	-	-	-	GQ165809
Nienokoue virus	ORF	-	-	-	NC_024299
Palm Creek virus	ORF	-	-	-	KC505248
Quang Binh virus	Genome	10,865	112	673	NC_012671

^a^ If multiple sequences have been deposited into the Genbank database, usually the Genbank accession number corresponding to the prototype isolate or longest sequence is shown; ^b^ Data not available.

The codon and dinucleotide usage preferences of cISFs are consistent with their apparent vertebrate-incompetent replication phenotype [79,80]. Vertebrates and invertebrates preferentially have certain codon and dinucleotide usage biases, and studies performed with RNA viruses have shown that their preferences often mimic those of their hosts [81,82,83,84]. Vertebrates display a strong under-representation of UpA and CpG, and over-representation of UpG and CpA. Mosquitoes also display a strong under-representation of UpA but have no bias for CpG depletion or for UpG and CpA excess [85]. A comparison of the dinucleotide usage preferences of representative viruses from the cISF, NKV and dual-host groups (CxFV, MODV and WNV) revealed that MODV and to a lesser extent WNV demonstrate a CpG decrease while CxFV has no bias against this dinucleotide [79]. All three viruses demonstrate an underutilization of UpA. Similar observations were reported when the dinucleotide usage preferences of CFAV, CxFV and KRV were compared to that of multiple NKV and dual-host flaviviruses [80].

**Figure 2:**
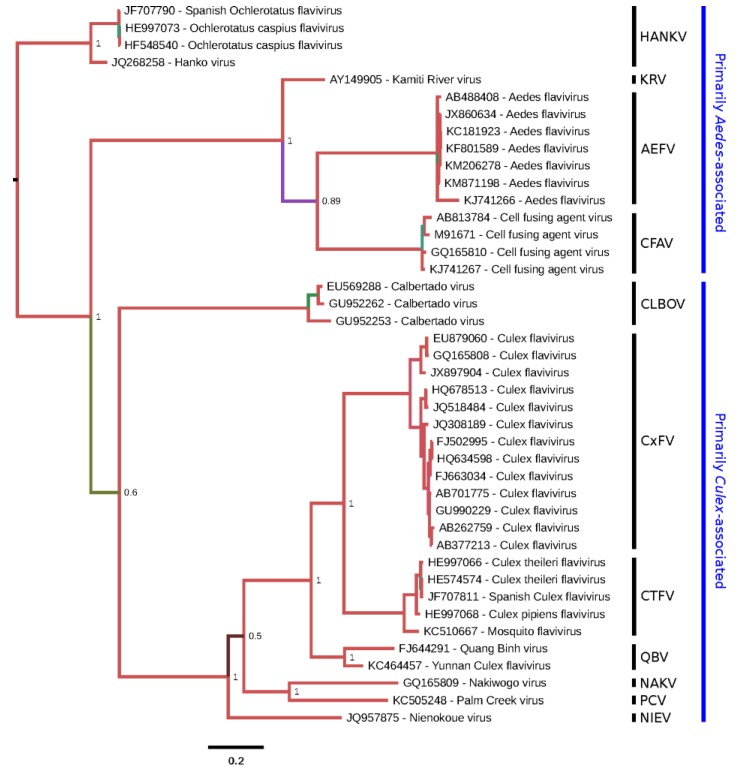
Phylogenetic tree for selected cISF partial NS5 sequences. A 795-nt region of NS5 corresponding to nt 8916-9710 of M91671.1 (CFAV) was used in order to include CLBOV, for which only partial NS5 sequences are available. The corresponding amino acid sequences were aligned with MUSCLE [14] and this amino acid alignment was used to guide a nucleotide sequence alignment. A maximum likelihood phylogenetic tree was estimated using the Bayesian Markov chain Monte Carlo method implemented in MrBayes version 3.2.3 [16] using the general time reversible (GTR) substitution model with gamma-distributed rate variation across sites and a proportion of invariable sites. Chains were run for 10 million generations, with the first 25% discarded as burn-in. The figure was produced using FigTree (http://tree.bio.ed.ac.uk/software/figtree/). Based on the full-genus tree (Figure 1), HANKV was selected as an outgroup to root the tree. Nodes are labelled with posterior probability values and poorly supported branches are also highlighted with alternative colors. Tips are labelled with isolate names as provided in original publications or, if unpublished, in sequence records. Species (as defined in this review) are grouped (vertical black bars) and annotated at right.

Classical ISFs are phylogenetically distinct from all other known flaviviruses (Figure 1). These viruses currently separate into two main clades (Figure 2). Clade 1 is composed of cISFs usually associated with *Aedes* spp. mosquitoes (AEFV, AGFV, CFAV and KRV). Subclade 2 contains *Culex*-associated viruses (CLBOV, CTFV CxFV, NIEV and QBV) in addition to NAKV and PCV, which were isolated from *Mansonia* and *Coquillettidia* spp. mosquitoes, respectively. HANKV is highly divergent from both clades 1 and 2 and may be regarded as forming a third clade. Although this virus has been detected in *Culex* spp. mosquitoes, it is more frequently associated with *Aedes* spp. mosquitoes [25,39,42]. 

### 2.6. Ribosomal Frameshifting

Programmed -1 ribosomal frameshifting (-1 PRF) is the process by which specific mRNA sequences induce a proportion of ribosomes to shift -1 nt and continue translating in the new reading frame [86]. PRF is utilized by many RNA viruses to control gene expression and to increase the number of protein products that can be expressed from a limited number of mRNA transcripts. The eukaryotic -1 frameshift site usually consists of a ‘slippery’ heptanucleotide fitting the motif X XXY YYZ (where XXX normally represents any three identical nucleotides although certain exceptions such as UCC, GGA, GUU and GGU also occur; YYY represents AAA or UUU; Z represents A, C or U; and spaces separate zero-frame codons), followed by a 5 to 9 nt ‘spacer’ region and then a stable RNA secondary structure such as a pseudoknot or stem-loop. 

**Figure 3:**
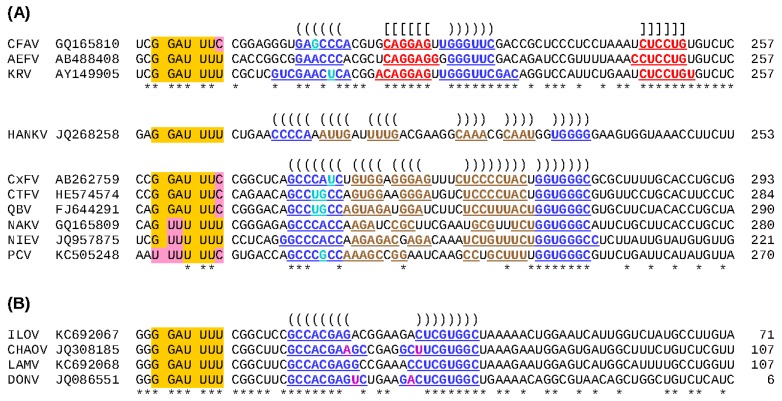
Predicted -1 frameshift sites in ISFs. (**A**) Apparently all cISFs contain a -1 PRF site just downstream of the predicted junction between the regions encoding NS1 and NS2A. Frameshifting results in translation of a long overlapping ORF, termed *fifo*. The ‘slippery’ heptanucleotide sequence at which the -1 nt shift occurs is highlighted in orange, with nucleotide variations highlighted in pink. Ribosomes that shift -1 nt read the last nucleotide of the heptanucleotide twice. Predicted frameshift stimulatory elements (an RNA pseudoknot structure in the CFAV clade and an RNA stem-loop structure in the HANKV and CxFV clades) are annotated: nucleotides predicted to be involved in base-pairing interactions are colored and underlined, and predicted base-pairings are indicated with “()”s and “[]”s (see also Figure 4). Conserved positions are indicated with “*”s. The length (in codons) of the *fifo* ORF in each sequence is indicated at right; (**B**) There is strong comparative genomic evidence that members of the dISF clade encompassing ILOV, CHAOV, LAMV and DONV contain a functionally utilized -1 PRF site towards the 3' end of the region encoding NS2B.

**Figure 4:**
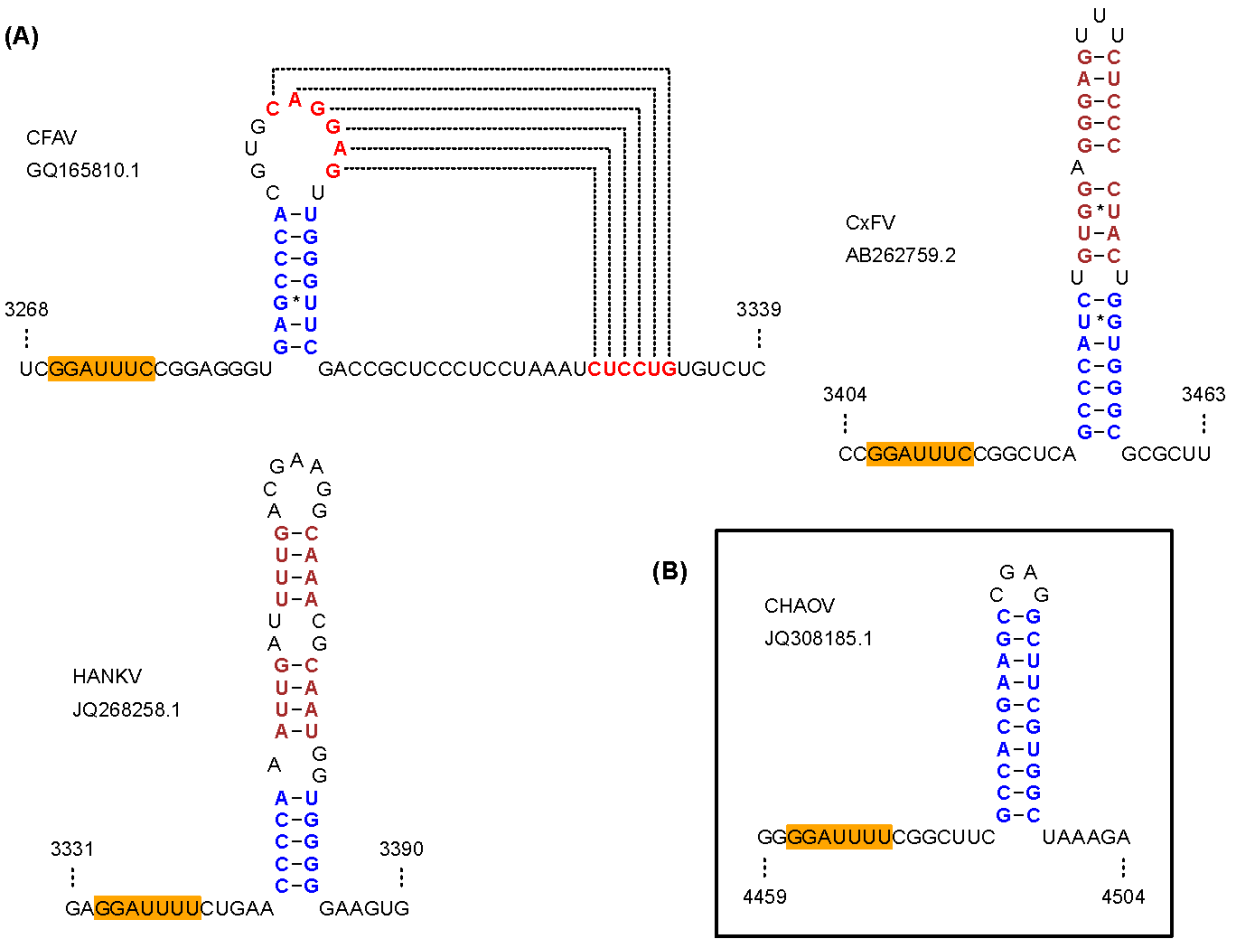
Predicted frameshift-stimulatory RNA structures in ISFs. (**A**) Frameshifting in cISFs is predicted to be stimulated by an RNA pseudoknot structure in the CFAV clade, and an RNA stem-loop structure in the CxFV and HANKV clades; (**B**) Frameshifting in the CHAOV clade of dISFs is predicted to be stimulated by an RNA stem-loop structure.

Previous studies have provided evidence that all known cISFs utilize -1 PRF to express a novel overlapping gene (designated *fifo*) in the NS2A-NS2B regions of their genomes [87]. However, CTFV, HANKV, NIEV and PCV had not been discovered when this conclusion was reached. To investigate whether -1 PRF is a universal feature of cISFs, the nucleotide sequences of all cISFs for which NS2A-NS2B data are available (as of 22 January 2015) were analyzed. All sequences were shown to possess a heptanucleotide sequence that conforms to the requirements of a ‘slippery’ -1 PRF motif (Figure 3A) and a downstream *fifo* ORF ranging from 221 (NIEV) to 293 (CxFV) codons. The exception to this is the laboratory-adapted isolate of CFAV; the *fifo* ORF of this isolate is disrupted by three premature termination codons suggesting that the gene is dispensable for *in vitro* replication. In addition to the slippery heptanucleotide frameshift site sequence, all cISF sequences were found to contain a potential stem-loop (CxFV and HANKV clades) or pseudoknot (CFAV clade) structure at the appropriate spacing downstream of the slippery heptanucleotide (Figure 3A and Figure 4A). It is interesting to note that this overlapping gene is unique to viruses in the cISF group; it is not encoded by the genomes of any other flaviviruses. Nevertheless, -1 PRF is utilized by various other flaviviruses. Apparently all viruses in the Japanese encephalitis (JE) serogroup, except for SLEV, utilize efficient -1 PRF to produce a larger NS1-related protein (NS1’) and to reduce synthesis of the 3'-encoded non-structural proteins relative to the proteins encoded upstream of the frameshift site [88,89,90,91]. -1 PRF has also been predicted to occur in many dISFs [87] (see Section 3f) and in Wesselsbron and Sepik viruses [92].

**a Table 3:** Predicted cleavage sites in the polyproteins of cISFs.

Junction	AEFV	CFAV	CxFV	CTFV	HANKV	KRV	NAKV	NIEV	PCV	QBV	Dual-Host Flaviviruses
Virion C/Anch	^b^ LEAQR↓SHSPV	^c^ LESRR↓TTGNP	^d^ LEAKR↓SAKNA	LEVRR↓SANNP	LEKER↓SHPRK	^e^ LEKQR↓SGPNL	LEKRR↓GVWSP	LEQRR↓GAQRG	LEKKR↓DGRAA	LENRR↓SANPL	After dibasic residues
C/prM	^b^ GLALS↓ETLRY	^j^ VLCGC↓VVIDM	^n^ MMVLG↓AVVID	VLCGC↓VIIDM	IVVTG↓LSIEL	^e^ GLCYG↓EMLRY	VGIFS↓LNVVD	MVTFA↓AVVDV	FGVMG↓VVVID	TLCGT↓MVIDM	Signalase-like cleavage
pr/M	^b^ PRKRR↓SSPQR	KREKR↓SREPP	^d^ KRERR↓VASTN	KRVKR↓APETP	ERETR↓QKVDD	^e^ VRRRR↓APQPQ	NRKQR↓SVKDE	RPVRR↓DVTPA	TRAKR↓VAPDG	KRVKR↓ATEQP	Furin
prM/E	^b^ NVVRA↓TSIEP	^j^ TTVKG↓EFVEP	^d^ TTVKG↓EFVEP	TTVKG↓EFVEP	NVVKG↓EFVEP	^e^ NVVKA↓SSIEP	TTVRG↓EFMEP	TTVSG↓EYLEP	TTVRG↓EYMEP	STVKG↓EFVEP	Signalase-like cleavage
E/NS1	^f^ RRVAG↓DIGCG	^c^ YYVRA↓DLGCG	^d^ VYTKA↓DVGCG	YFARA↓DVGCG	VYVKA↓DVGCG	^e^ RSVSA↓DVGCG	YTVRA↓DFGCG	YYVRA↓DVGCG	YFVRA↓DFGCG	YYTRA↓DVGCG	Signalase-like cleavage
NS1/NS2A	^b^ GKADA↓TADFH	^c^ GKANA↓QSDFR	° PPVEG↓SYPDF	PGTGA↓FPDFQ	YRVPS↓TNAED	^e^ GKAHA↓CSDFR	PPSGA↓EKLQQ	GGAEA↓TQSFF	PMGET↓AKIQN	PGAEA↓LLQDF	Signalase-like cleavage
NS2A/NS2B	^g^ KSSYR↓TSGRS	^k^ RNGYR↓DSGAN	^p^ RSGLR↓ASRRS	KSGLR↓ASKSS	RSGYR↓ALCSS	^s^ KNGYR↓DYGAS	ASGLR↓KPRPH	KSGLR↓SITSW	GDGLR↓APRPH	KSGLR↓ASKRS	After dibasic residues
NS2B/NS3	^b^ NEHCR↓SDDLL	^c^ TASNR↓SDDLL	^q^ VSVFR↓SNEVN	STAYR↓AGVND	TNAFR↓SDELI	^e^ SEQNR↓SDDLL	EFAQR↓SSSEL	STAQR↓SDLLL	AMSQR↓ANSEL	TSNRR↓SGVND	After dibasic residues
NS3/NS4A	^h^ YINTR↓SSASL	^l^ YMNCR↓GGPTL	^r^ YLKQR↓SNFNF	FLKQR↓SGANF	YMGTR↓SFLSV	^t^ YLNCR↓SSQTF	FLKQR↓SVLPF	FLKQR↓SLFID	FLKQR↓SLYFD	FLKQR↓SVLNF	After dibasic residues
NS4A/2K	AAGNR↓SYLDS	SIGNR↓SYMDS	NNVHR↓AYTTD	NNVHR↓AYTGD	SAGQR↓SYVDI	AIGNR↓SYMDS	GGSQR↓GILDS	ANSQR↓GFAEN	GGSQR↓GVLDS	TNVHR↓AYTGD	After dibasic residues
2K/NS4B	^b^ CSVLA↓WEMRL	^c^ CGVLA↓WEMRM	^d^ MGVVA↓WEMDL	MGVVA↓WELNL	IGVIC↓WELRL	^e^ CGVLA↓WEMRL	IGIAA↓WELQL	SAVVA↓WELNL	IGVTA↓WELEL	MGIVA↓WELEL	Signalase-like cleavage
NS4B/NS5	^i^ FSKFR↓ALEKS	^m^ FNQFR↓ALEKS	^d^RMALR↓SLVKT	RGGLR↓SLVKT	NITTR↓SLEKS	^u^ FNQFR↓ALEKS	RLSVR↓SLVKS	LDMRR↓SLMKT	RLGVR↓SLVKS	RLATR↓SLVKT	After dibasic residues

^a^ Genbank Accession numbers for the sequences used in this analysis are listed in Table 2; ^b^ Consistent with the AEFV polyprotein cleavage sites proposed by [19]; ^c^ Consistent with the CFAV polyprotein cleavage sites proposed by [18]; ^d^ Consistent with the CxFV polyprotein cleavage sites proposed by [31]; ^e^ Consistent with the KRV polyprotein cleavage sites proposed by [44]; ^f^ 1 residue downstream of the AEFV E/NS1 cleavage site proposed by [19]; ^g^ 25 residues upstream of the AEFV NS2A/NS2B cleavage site proposed by [19]; ^h^ 5 residues downstream of the AEFV NS3/NS4A cleavage site proposed by [19]; ^i^ 1 residue upstream of the AEFV NS4B/NS5 cleavage site proposed by [19]; ^j^ Cleavage at this junction was experimentally verified by amino-terminal sequencing [18]; ^k^ 25 residues upstream of the CFAV NS2A/NS2B cleavage site proposed by [18]; ^l^ 10 residues downstream of the CFAV NS3/NS4A cleavage site proposed by [18]; ^m^ One residue upstream of the CFAV NS4B/NS5 cleavage site proposed by [18]; ^n^ 1 residue upstream of the CxFV C/prM cleavage site proposed by [31]; ° 24 residues downstream of the CxFV NS1/NS2A cleavage site proposed by [31] but consistent with [87]; ^p^ 4 residues upstream of the CxFV NS2A/NS2B cleavage site proposed by [31]; ^q^ 15 residues downstream of the CxFV NS2B/NS3 cleavage site proposed by [31]; ^r^ 10 residues downstream of the CxFV NS3/NS4A cleavage site proposed by [31]; ^s^ 25 residues upstream of the KRV NS2A/NS2B cleavage site proposed by [44]; ^t^ 10 residues downstream of the KRV NS3/NS4A cleavage site proposed by [44]; ^u^ 1 residue upstream of the KRV NS4B/NS5 cleavage site proposed by [44].

Immunoflorescence assays performed using polyclonal antibodies raised against two predicted CxFV FIFO antigens detected a protein product in CxFV-infected, but not mock-infected, C6/36 cells [87]. However, bands of the expected size were not detected when lysates from CxFV-infected cells were analyzed by Western blot using these same antibodies. Thus, it is not known whether *fifo* is expressed as a frameshift fusion simply with the N-terminal few amino acids of NS2A (*i.e.*, NS2A^N^-FIFO) or, as in the case of the JE serogroup NS1' protein, as a fusion also with NS1 (*i.e.*, NS1-NS2A^N^-FIFO). It is also possible that the fusion products are internally cleaved. Additional work is needed to investigate the expression and functional relevance of the *fifo* product in cISFs. 

### 2.7. Predicted Polyprotein Cleavage Sites

The predicted proteolytic cleavage sites of all cISFs for which complete polyprotein ORF data are available are shown in Table 3. For the most part, these sites conform to the rules established for dual-host flaviviruses although there are some exceptions. Studies performed with dual-host flaviviruses have revealed that a host signal peptidase mediates cleavage between C/prM, prM/E, E/NS1 and 2K/NS4B and that these junctions typically conform to predicted signalase cleavage sites [93]. Similar sites were identified at the predicted C/prM, prM/E, E/NS1 and 2K/NS4B junctions of most cISFs. The NS1/NS2A cleavage site of dual-host flaviviruses is signalase-like with respect to the '-1, -3' rule, but an upstream hydrophobic domain is absent. Previous work has demonstrated that, at least in DENV, cleavage requires translation of substantial parts of NS2A and it has been proposed that the hydrophobic domains in NS2A lead to a conformation that presents the NS1/NS2A cleavage site to an endoplasmic reticulum-resident host protease that may be signalase [94]. In the dual-host flaviviruses, NS1/NS2A cleavage usually occurs after a Val-X-Ala site. Although the predicted cISFs cleavage sites generally lack one or other of these residues, they are still compatible with the signalase '-1, -3' rule [95]. In dual-host flaviviruses, the cellular protease furin cleaves prM to generate the mature form of the protein [93,96]. Furin normally cleaves after the motif Arg-X-Lys/Arg-Arg but cleavage can also occur after Arg-X-X-Arg [97]. As for dual-host flaviviruses, the predicted pr/M junction of every cISF is preceded by RXKR or RXRR, except for HANKV and NAKV which contain only the minimal furin cleavage site RXXR.

The virion C/anchor, NS2A/NS2B, NS2B/NS3, NS3/NS4A, NS4A/2K and NS4B/NS5 junctions of dual-host flaviviruses are cleaved by the viral NS2B/NS3 serine protease, which normally cleaves after two basic amino acid residues (KR, RR, RK) or sometimes after QR at the P_2_ and P_1_ positions, followed by a small amino acid (G, A or S) at the P'_1_ position [93,98,99]. The corresponding cISF cleavage sites are not always obvious and frequently appear to deviate from these motifs. It should be noted that only two cISF cleavage sites have been experimentally determined (viz. CFAV anchorC/prM and prM/E) both of which are signalase rather than viral protease cleavage sites [18]. Alignment between cISF and dual-host or dISF flavivirus sequences at the NS2B/NS3, NS3/NS4A, NS4A/2K and NS4B/NS5 junctions suggests that cleavage in cISFs occurs between R at the P_1_ position and G, A or S at the P'_1_ position, but there seems to be substantial flexibility at the P_2_ position (Table 3). The exact cleavage site at the virion C/anchor junction was difficult to predict due to a cluster of basic amino acids; most cISFs contain a potential Arg-Gly/Ala/Ser (P_1_-P'_1_) cleavage site at the C-terminal end of the cluster of basic residues, while a few may use alternative motifs in this region. Prediction of the NS2A/NS2B cleavage site in cISFs was particularly problematic: while some species (e.g., CxFV) contain several Arg-Gly/Ala/Ser (P_1_-P'_1_) motifs in the critical region between two predicted transmembrane regions (aligning to the corresponding NS2A/NS2B junction in dual-host and dISF flaviviruses), other species contained no such motifs in this region. A conserved Arg residue was annotated as a potential cleavage site in Table 3, notwithstanding that in CFAV, KRV, AEFV and NAKV it is followed by Asp, Asp, Thr and Lys, respectively, while in several species (including AEFV) there are closely spaced alternative cleavage sites. It should be noted that, due to the additional constraints imposed on this sequence region in the cISFs as a result of the overlapping *fifo* ORF, it is possible that NS2A/NS2B cleavage in the cISFs may have evolved to take place at a somewhat different location than we have inferred from comparison to other flavivirus sequences.

In some instances, the predicted cleavage sites listed in Table 3 are different from those reported by others. For example, the NS2A/NS2B cleavage sites that we proposed for AEFV, CFAV and KRV are located 4 to 25 residues upstream of the sites previously proposed for these viruses [18,19,31,44]. Our predicted NS4B/NS5 cleavage sites are located one residue upstream of the sites originally proposed for AEFV, CFAV and KRV [18,19,44]. Our analysis was performed using additional cISF sequences, thus facilitating the identification of conserved sites. Nevertheless, our data are subjective and experimental data (e.g., amino acid sequencing) are needed to conclusively identify the cleavage sites in the polyproteins of cISFs. 

## 3. Dual-Host Affiliated Insect-Specific Flaviviruses

### 3.1. Discovery, Geographic Distribution and Natural Host Range

Dual-host affiliated ISFs are not as well characterized as cISFs. The first dISF reported in the literature was Nounané virus (NOUV) after its isolation from *Uranotaenia mashonaensis* in Côte d'Ivoire in 2004 [100] (Table 4). At least eight other dISFs have since been discovered: Barkedji virus (BJV) in Senegal (Genbank Accession No. EU078325) and Israel [101], Chaoyang virus (CHAOV) in South Korea [102,103] and China [104], Donggang virus (DONV) in China (Genbank Accession No. NC_016997), Ilomantsi virus (ILOV) in Finland [105], Lammi virus (LAMV) in Finland [106], Marisma mosquito virus (MMV) in Spain [39] and Italy [107], Nanay virus (NANV) in Peru [108] and Nhumirim virus (NHUV) in Brazil [109]. Evidence indicates that another dISF may occur in Kenya [110]. A region of the NS5 gene of the virus was sequenced and shown to have greatest (77%) nucleotide identity with the corresponding region of CHAOV. The sequence has not been deposited into the Genbank database and therefore cannot be compared with those of the more recently discovered dISFs in order to establish whether it is indeed a novel virus. Collectively, dISFs have been isolated from or detected in at least 12 species and five genera of mosquitoes (Table 4). Dual-host affiliated ISFs have not been isolated from or detected in sandflies, midges or any other non-mosquito dipterans. It is unclear whether dISFs truly have a mosquito-restricted host range because other dipterans are rarely screened for these viruses. Additional work should be done to address this issue.

**Table 4:** Geographic distribution and natural host range of dual-host affiliated insect-specific flaviviruses.

Virus	Isolate Available	Geographic Distribution	Natural Host Range	References
Barkedji virus (BJV)	No	Senegal (date not reported), Israel (2011)	*Cx. perexiguus*	[101], (Genbank Accession No. EU078325)
Chaoyang virus (CHAOV)	Yes	China (2008), South Korea (2003)	*Ae. vexans, Ae. albopictus, Ae. bekkui, Armigeres subalbatus, Cx. pipiens*	[102,103,104,111]
Donggang virus (DONV)	^a^ Yes	China (2009)	*Aedes spp.*	(Genbank Accession No. NC_016997)
Ilomantsi virus (ILOV)	Yes	Finland (2007)	Most likely *Oc. riparius* and/or *Anopheles* spp.	[105]
Lammi virus (LAMV)	Yes	Finland (2004)	*Ae. cinereus*	[106]
Marisma mosquito virus (MMV)	Yes	Spain (2003), Italy (2011)	*Ae. caspius*	[39,107]
Nanay virus (NANV)	Yes	Peru (2009)	*Culex (Melanoconion) ocossa*	[108]
Nhumirim virus (NHUV)	Yes	Brazil (2010)	*Cx. chidesteri*	[109]
Nounané virus (NOUV)	Yes	Côte d'Ivoire (2004)	*Uranotaenia mashonaensis*	[100]

^a^ Information provided in the Genbank database implies that an isolate is available for DONV.

### 3.2. In Vitro and in Vivo Replication Potential in Vertebrates and Arthropods

By definition, dual-host affiliated ISFs have not been isolated from any vertebrates in nature and data from laboratory experiments indicate that they possess a vertebrate-incompetent replication phenotype. Suckling mice intracerebrally inoculated with ILOV, LAMV or NANV displayed no signs of illness and viral RNA was not detected in brains harvested from these animals [105,106,108]. The most comprehensive *in vitro* experiments were performed by Huhtamo and colleagues who demonstrated that LAMV does not replicate in avian (primary chicken), canine (MDCK), hamster (BHK-21), human (SH-SY5Y, Hep, MRC-5, SW13, HEK293), monkey (Vero, BGM, MA104), mouse (Neuro 2A, L929), porcine (PK-15), snake or toad (XTC) cells [105,106]. Selected mammalian cells were also incubated at lower temperatures (27 °C and 33 °C) and the virus was still unable to replicate. The replicative abilities of several other dISFs have also been assessed in multiple vertebrate cell lines including ILOV which could not replicate in canine, hamster, monkey, mouse, human, snake or toad cells [105] and NOUV which could not replicate in avian, hamster, human, monkey or porcine cells [100]. It has been suggested that transient replication of MMV occurs in Vero and BHK-21 cells [39]. Cytopathic effect (CPE) was observed and viral RNA was detected at 5 to 7 days post-inoculation. However, neither CPE nor viral RNA was evident after the first passage and thus, the cells could not support prolonged MMV replication. One limitation of this study is that the authors did not show an increase in viral genome copy number by quantitative RT-PCR. The experiments were performed using conventional RT-PCR and therefore it is not clear whether the detected viral RNA was due to active virus replication or the presence of viral RNA in the original inoculum. The CPE may have been caused by an unrecognized virus in the original inoculum. 

The replicative abilities of several dISFs have been assessed in multiple arthropod cell lines. LAMV and ILOV replicate efficiently in *Ae. albopictus* (C6/36 and AA23) and *Ae. aegypti* (AE and A20) cells [105]. NHUV replicates efficiently in *Ae. albopictus* (C6/36 and C7/10) and *Cx. quinquefasciatus* cells but not *Ixodes scapularis* tick (ISE6) cells [79]. CPE was first observed in NHUV-infected C6/36 cells at 3 and 6 days p.i. after the initial inoculation and second passage, respectively. MMV, NANV and NOUV are also known to infect C6/36 cells, with CPE observed in cells inoculated with the latter two viruses [39,100,108]. CHAOV also replicates in C6/36 cells but there are conflicting reports as to whether or not it induces CPE [102,111]. *In vivo* experiments have not been performed to assess the infection, replication and dissemination abilities of dISFs in mosquitoes or any other arthropods.

### 3.3. Transmission

The mechanism by which dISFs are maintained in nature is not known. Their phylogenetic placement with mosquito/vertebrate flaviviruses indicates that these viruses are (1) dual-host viruses with an unidentified vertebrate host or (2) insect-specific viruses that recently lost the ability to infect vertebrates. Given that the replicative abilities of several dISFs have been assessed in numerous vertebrate cell lines with none of the cell lines able to support prolonged virus replication, the second explanation appears more likely. However, there are no other experimental data to support this. All dISFs have been isolated from adult female mosquitoes or from mosquitoes of unspecified genders and life stages. To shed light on whether dISFs are maintained by vertical transmission, immature and adult male mosquitoes need to be screened for these viruses. Moreover, *in vivo* mosquito infections similar to those described in 2c need to be performed to determine the importance of vertical, horizontal and mechanical transmission in dISF maintenance. 

### 3.4. Competitive Interaction between dISFs and Dual-Host Flaviviruses

Data on the ability of dISFs to suppress the replication of dual-host ISFs in mosquito cells are limited. *In vivo* experiments have not been performed and only one study has assessed the *in vitro* superinfection exclusion potential of a dISF [79]. Briefly, prior or concurrent inoculation of mosquito (C6/36) cells with NHUV significantly suppressed the replication of WNV, JEV and SLEV [79]. The effect was most pronounced in cells inoculated with WNV and SLEV; the peak viral titers of these viruses were reduced 10^6^- and 10^4^-fold, respectively. JEV exhibited an 80-fold reduction in peak titer. Titers were significantly reduced as early as 1 day p.i. (WNV and JEV) and 2 days p.i. (SLEV). 

### 3.5. Genome Sequencing and Phylogeny

Complete genomic sequences are available for three dISFs: CHAOV, DONV and NHUV (Table 5). Complete polyprotein ORF sequences are available for ILOV, LAMV and NOUV and nearly all of the BJV ORF has also been sequenced. Partial E and/or NS5 sequences are available for MMV and NANV. The 5’UTRs of CHAOV, DONV and NHUV are similar in length (99, 113 and 102 nt, respectively) to those of most other flaviviruses. The 3’ UTR of NHUV (451 nt) is within the size range usually reported for flaviviruses while the 3’ UTRs of CHAOV and DONV (326 and 343 nt, respectively) are slightly shorter than expected. 

**Table 5:** Summary of sequence data available for dual–host affiliated insect-specific flaviviruses.

Virus	Sequence Data Available	Length of Genome (nt)	Length of 5’ UTR (nt)	Length of 3’ UTR (nt)	^a^ Genbank Accession No.
Barkedji virus	Almost entire ORF	^b^ -	-	-	KC496020
Chaoyang virus	Genome	10,733	99	326	NC_017086
Donggang virus	Genome	10,791	113	343	NC_016997
Ilomantsi virus	ORF	-	-	-	NC_024805
Lammi virus	ORF	-	-	-	KC692068
Marisma mosquito virus	Partial NS5	-	-	-	JN603190
Nanay virus	Partial E and NS5	-	-	-	JX627335
Nhumirim virus	Genome	10,891	102	451	NC_024017
Nounané virus	ORF	-	-	-	EU159426

^a^ If multiple sequences have been deposited into the Genbank database, the accession number corresponding to the prototype isolate and/or longest sequence is shown in most instances; ^b^ Data not available.

**Figure 5:**
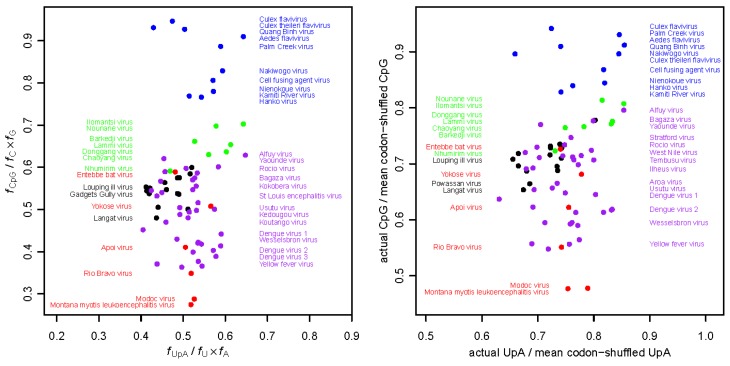
Relative UpA and CpG frequencies in different flavivirus species. UpA and CpG frequencies were calculated in two different ways. (**A**) In each sequence, the numbers of UpA and CpG dinucleotides, and A, C, G and U mononucleotides, were counted. Dinucleotide frequencies, *f*_XpY_, were expressed relative to their expected frequencies, *f*_X_ x *f*_Y_, in the absence of selection; (**B**) Since codon usage reflects dinucleotide bias but can also be subject to other selective pressures (e.g., for translational speed or accuracy) that, due to co-evolution of dinucleotide and codon preferences in the host, may lead to the same dinucleotide biases, we also calculated dinucleotide biases independent of codon (and amino acid) usage. To factor out codon and amino acid usage, 1000 shuffled ORF sequences were generated for each virus sequence. In each shuffled sequence, the original amino acid sequence and the original total numbers of each of the 61 codons were maintained, but synonymous codons were randomly shuffled between the different sites where the corresponding amino acid is used in the original sequence. Then the UpA and CpG frequencies in the original sequence were expressed relative to their mean frequencies in the codon-shuffled sequences. Because codon usage is factored out, the UpA and CpG relative frequencies tend to be less extreme in (**B**) compared to (**A**). Since many sequences lack complete UTRs, for consistency, both analyses of all species were restricted to the polyprotein ORF. Each point represents a single flavivirus sequence. Points and selected species names are color-coded as follows: cISFs—blue; dISFs—green; NKV flaviviruses—red; mosquito/vertebrate flaviviruses—purple; tick/vertebrate flaviviruses—black. GenBank accession numbers are the same as those used in Figure 1.

A comparison of the dinucleotide usage preferences of NHUV to representative flaviviruses from the cISF, NKV and dual-host groups (CxFV, MODV and WNV, respectively) revealed that the CpG usage of NHUV was similar to that of WNV [73] whereas, as mentioned in Section 2.5, CxFV has no strong bias against this dinucleotide. These findings could indicate that NHUV is not a mosquito-specific virus but instead a dual-host virus with a yet-to-be-determined vertebrate host. Another explanation is that dISFs lost their ability to replicate in vertebrates relatively recently and still possess dinucleotide usage preferences that partly reflect the dual-host precursor viruses from which they evolved. In support of this interpretation, on average dISFs have higher CpG usage than dual-host flaviviruses though lower usage than cISFs (Figure 5).

As already noted, dISFs phylogenically affiliate with mosquito/vertebrate flaviviruses (Figure 1). 

These viruses cluster within the mosquito-borne flavivirus clade. CHAOV, LAMV, DONV and ILOV form a distinct clade. NHUV, BJV and NOUV form another clade. However it is not clear that these viruses can be grouped together into a single monophyletic clade. Thus, if dISFs are truly insect-specific viruses that lost the ability to infect vertebrates, this topological arrangement suggests that loss of vertebrate host happened separately for each of these two clades.

### 3.6. Ribosomal Frameshifting

For members of the dISF clade comprising CHAOV, LAMV, DONV and ILOV, -1 PRF has been predicted to occur within the NS2B-encoding region of the genome [87]. A slippery heptanucleotide and 3' predicted stem-loop structure are phylogenetically conserved within the group [105] (Figure 3B and Figure 4B) and the sequence elements have been shown to stimulate frameshifting in a reporter construct [87]. Frameshifting would result in an alternative version of NS2B with a different C-terminal tail encoded by the -1 reading frame.

**a Table 6:** Predicted cleavage sites in the polyproteins of dISFs.

Junction	BJV	CHAOV	DONV	ILOV	LAMV	NHUV	NOUV	Dual-Host Flaviviruses
Virion C/Anch	^b^ KTSKR↓GLQQS	RKAKR↓SVTTP	RPNRR↓SAGSN	QKTRR↓SVDTV	KNGKR↓SKTEI	^c^ RRARR↓GMGIP	^d^ VSKRR↓GSASL	After dibasic residues
C/prM	^b^ TMAAC↓ATLGM	CMAYG↓ATRFT	GTAMA↓ATSMT	VAVIA↓TTVTT	GTAMA↓ASMFT	^b^ TMVAC↓VTVGT	^d^ GVASA↓VTFTT	Signalase-like cleavage
pr/M	^b^ RRSKR↓SVAIA	RRSRR↓SVALA	RRSRR↓SIMIP	RRSRR↓SIALA	RRGKR↓SVALA	^b^ RRSRR↓SVALS	^d^ QRSRR↓SVGIS	Furin
prM/E	^b^ APAYS↓LHCSR	GPAYS↓LQCID	APVYG↓SQCSG	APVYG↓HHCSG	GPAYS↓LQCVD	^b^ APAYS↓THCVR	^d^ IPAYS↓MKCIG	Signalase-like cleavage
E/NS1	^b^ TTVAG↓DVGCN	TVGVS↓EIGCS	TNAVS↓EVGCS	SAAAS↓EVGCS	TVALS↓EVGCS	^b^ TSAHA↓EVGCS	^c^ TSVSA↓ELGCS	Signalase-like cleavage
NS1/NS2A	^b^ SWTTA↓GNATG	SKVSA↓GTFQG	ARVSA↓GAVHG	ARVSA↓GLVAG	SKVSA↓GTFQG	^b^ SWVTA↓GQMTG	^e^ SWVSA↓GEPMV	Signalase-like cleavage
NS2A/NS2B	^b^ GSGKR↓SVSMG	SSGKR↓SWPAG	KHGKR↓SWPAG	RNGRR↓SWPAG	TSGKR↓SWPAG	^b^ KSGKR↓SVSMG	^d^ KTTKR↓SVPQS	After dibasic residues
NS2B/NS3	^b^ KGTQK↓AGAMW	KSGRR↓GTVLW	KHDRR↓GGVLW	RTAKR↓GGVLW	KSGRR↓GTVLW	^b^ SATQR↓AGAMW	^d^ ENRKR↓SNDTP	After dibasic residues
NS3/NS4A	^b^ AEGRR↓GASDI	AEGRR↓SYVPI	AEGRR↓SYMPI	AEGKR↓SAVQL	AEGRR↓SYVPL	^b^ AEGRR↓GAMDL	^d^ AGGKR↓SAVDL	After dibasic residues
NS4A/2K	AEKQR↓SAIDN	PGSQR↓SVQDN	PGNQR↓SIQDN	AGGQR↓SIADN	PGSQR↓SVQDN	AEKQR↓SALDN	^d^ EGKQR↓SMVDN	After dibasic residues
2K/NS4B	^b^ LAVTA↓NEKGL	ALIAA↓NETGL	GGIAA↓NEMGM	SLIAA↓NETGL	ALIAA↓NETGL	^b^ LMIAA↓NEKGL	^d^ GAVAA↓NEYGM	Signalase-like cleavage
NS4B/NS5	^b^ KSARK↓GTPGG	GVPRR↓GVTIS	QPSRR↓GKKVE	TTPRR↓GRRVN	GVPRR↓GMTIC	^b^ KSARR↓GTPGG	^f^ VVTRK↓GTAGG	After dibasic residues

^a^ Genbank Accession numbers for the sequences used in this analysis are listed in Table 5; ^b^ Consistent with the BJV and NHUV polyprotein cleavage sites proposed by [79]; ^c^ 3 residues downstream of the NHUV virion C/anch C cleavage sites proposed by [79]; ^d^ Consistent with the NOUV polyprotein cleavage sites proposed by [100]; ^e^ 15 residues upstream of the NOUV NS1/NS2A cleavage site proposed by [100]; ^f^ 30 residues upstream of the NOUV NS4B/NS5 cleavage site proposed by [100].

In NOUV, a potential frameshift site (U UUU UUA with a 3'-adjacent predicted stem-loop structure) was identified in the NS2A-encoding region, and the sequence was shown to stimulate frameshifting in a reporter construct [87]. Frameshifting at this site would yield a truncated version of NS2A. The site is not conserved in BJV or NHUV, but a different potential frameshift site (again U UUU UUA with a 3'-adjacent predicted stem-loop structure) occurs in NHUV at a site around 25 codons upstream (again within NS2A). Between these two sites, BJV has a potentially slip-prone U CCU UUU sequence, but only a weak 3'-adjacent stem-loop structure was predicted. It remains unclear whether the NOUV, NHUV and BJV sites are chance features or functionally relevant -1 PRF sites.

### 3.7. Predicted Polyprotein Cleavage Sites

The predicted proteolytic cleavage sites in the polyproteins of all dISFs for which complete or almost complete ORF data are available are shown in Table 6. It is interesting to note that the predicted cleavage sites of these viruses more closely resemble those of dual-host flaviviruses than those of the cISFs. For instance, nearly all dISFs appear to utilize a Val-X-Ala cleavage site at the NS1/NS2A junction consistent with dual-host flaviviruses [93]. The exception to this rule is BJV which has Thr instead of Val at the -3 position. In contrast, cISFs tend to use more varied signalase-like motifs at this site. Dual-host flaviviruses usually contain residues KR, RR, RK or QR immediately upstream, and a G, A or S residue immediately downstream of the virion C/anchor, pr/M, NS2A/NS2B, NS2B/NS3, NS3/NS4A, NS4A/2K and NS4B/NS5 cleavage sites [93]. Every dISF conforms to this rule at all of the aforementioned junctions with the exception of BJV at the NS2B/NS3 junction where cleavage is predicted to occur between QK and A. 

## 4. Closing Remarks and Future Research

Historically, ISFs have received limited attention due to the assumption that they have minimal, if any, impact on human health by virtue of their apparent insect-specific phenotype. However, there are several important reasons why these viruses should not be neglected. Comparative studies on insect-specific and dual-host flaviviruses will provide unique insight into why some flaviviruses are major human pathogens while others possess a vertebrate-incompetent replication phenotype. For instance, the generation and subsequent characterization of chimeric viruses of insect-specific and dual-host flaviviruses will result in the identification of the genetic elements that modulate the differential host ranges, transmission cycles and tropisms of these viruses. The characterization of chimeras of NKV and dual-host flaviviruses will build upon these studies via the identification of elements that preclude flaviviral replication in the mosquito host. Several groups have begun to address this issue through the characterization of NKV and dual-host flaviviral chimeras [112,113,114,115] but no ISF/dual-host flaviviral chimeras have been described. 

Some ISFs possess the ability to enhance or suppress the replication of dual-host flaviviruses in co-infected mosquitoes; thus, these viruses warrant further investigation due to the indirect impact that they have on human health. Another reason why it is important to increase our understanding of ISFs is because these viruses have the potential to be used to control mosquito populations and impede disease transmission. Genes that are essential for mosquito development, reproduction or longevity, or dual-host flaviviral infection, dissemination or transmission, could be silenced via the release of recombinant ISFs engineered to express specific genes or sequences. Only one ISF infectious cDNA clone has been described [116] and most ISFs have not been fully sequenced. The availability of additional genomic sequence data and infectious cDNA clones would assist in this area of research. However, caution is required if recombinant ISFs are to be released in the environment because, as noted above, some cISFs possess the ability to enhance the replication of dual-host flaviviruses in coinfected mosquitoes. A final reason why ISFs should be further investigated is because they may have the potential to evolve into major human pathogens. 

The identification and characterization of ISFs is dependent upon the availability of reliable detection tools, including ISF-specific monoclonal antibodies (MAbs). ISF-specific MAbs are available for one virus [46] although some polyclonal antibodies raised against dual-host flaviviruses cross-react with ISFs [37,105,106,108]. The production of additional ISF-specific MAbs would assist in the future identification and characterization of these viruses. Future research should also investigate the mechanism(s) by which dISFs are maintained in nature. Although the significance of vertical (*i.e.*, transovarial and venereal) and mechanical transmission in cISF persistence has been assessed, similar studies have not been done with dISFs. The screening of immature or adult male mosquitoes could easily be incorporated into dISF identification and surveillance studies, and the resulting data would provide important insight into the role, if any, of vertical transmission in dISF persistence. Research should also be done to investigate the significance of the overlapping *fifo* ORF uniquely encoded in the NS2A-NS2B region of the cISF genome, and shorter predicted ORFs in similar regions of some dISF genomes. Because *fifo* is not encoded by any vertebrate-infecting flaviviruses, this gene could have a key role in the apparent ability of ISFs to be maintained in nature in the absence of a vertebrate host. For instance, *fifo* could have a pivotal role in TOT. Research is also needed to determine whether ISFs infect non-mosquito dipterans. 

ISFs are presumably under-represented in the literature and sequence databases compared to their vertebrate-infecting counterparts. One explanation for this presumed under-representation is because some ISFs cause minimal (if any) CPE in mosquito cells and none possess the capacity to replicate in mammalian cells. Therefore, ISFs could easily remain undetected in virus surveillance studies in which mosquitoes are screened for viruses by suckling mouse brain inoculation or by virus isolation in Vero or C6/36 cells. Many dual-host flaviviruses were initially discovered as a result of their association with outbreaks of human disease. In contrast, ISFs are not associated with disease outbreaks due to their vertebrate-incompetent replication phenotype. 

To summarize, additional research on ISFs is desirable because it will provide unique insight into medically important flaviviruses and has the potential to lead to the development of efficient vector and disease control strategies. Despite the dramatic increase in the number of ISFs identified in recent years, as well as the sudden increase in ISF-related research, it is likely that many undiscovered ISFs occur in nature and there are clearly many areas of ISF research that have not been pursued. 
